# Identification of serum cytokine clusters associated with outcomes in ovarian clear cell carcinoma

**DOI:** 10.1038/s41598-020-75536-1

**Published:** 2020-10-28

**Authors:** Akira Yabuno, Hirokazu Matsushita, Tetsutaro Hamano, Tuan Zea Tan, Daisuke Shintani, Nao Fujieda, David S. P. Tan, Ruby Yun-Ju Huang, Keiichi Fujiwara, Kazuhiro Kakimi, Kosei Hasegawa

**Affiliations:** 1 Department of Gynecologic Oncology, Saitama Medical University International Medical Center, 1397-1 Yamane, Hidaka-shi, Saitama, 350-1298 Japan; 2 Department of Immunotherapeutics, The University of Tokyo Hospital, 7-3-1 Hongo, Bunkyo-ku, Tokyo, 113-8655 Japan; 3 Cancer Science Institute of Singapore, National University of Singapore, Singapore, Singapore; 4 Department of Medicine, Yong Loo Lin School of Medicine, National University of Singapore, Singapore, Singapore; 5 National University Cancer Institute, Singapore, National University Hospital, Singapore, Singapore; 6 School of Medicine, College of Medicine, National Taiwan University, Taipei, Taiwan

**Keywords:** Ovarian cancer, Cytokines

## Abstract

Serum cytokine and chemokine networks may reflect the complex systemic immunological interactions in cancer patients. Studying groups of cytokines and their networks may help to understand their clinical biology. A total of 178 cases of ovarian cancer were analyzed in this study, including 73 high-grade serous (HGSC), 66 clear cell (CCC) and 39 endometrioid carcinomas. Suspension cytokine arrays were performed with the patients’ sera taken before the primary surgery. Associations between each cytokine and clinicopathological factors were analyzed in all patients using multivariate linear regression models, and cluster analyses were performed for each histotype. In the multivariate analyses, twelve of 27 cytokines were correlated with histotypes. Cluster analyses in each histotype revealed 2 cytokine signatures S1 and S2 in HGSC, and similarly C1 and C2 in CCC. Twenty-two of 27 cytokines were commonly clustered in HGSC and CCC. Signature S1 and C1 included IL-2,6,8,15, chemokines and angiogenic factors, whereas signature S2 and C2 included IL-4,5,9,10,13, TNF-α and G-CSF. Four subgroups based on a high or low level for each signature were identified, and this cluster-based classification demonstrated significantly different progression-free and overall survivals for CCC patients (*P* = 0.00097 and *P* = 0.017).

## Introduction

Ovarian cancer is the most lethal disease among gynecological malignancies. In 2018, it was the 8th leading cause of cancer incidence and the 8th leading cause of cancer-associated mortality among females all over the world^1^. Epithelial ovarian cancers (EOC) are the most common, accounting for 90% of all cases^2^. Although 65% of EOC are diagnosed at stage III or IV, the incidence and stage at diagnosis vary by histotype, age and race/ethnicity. High-grade serous carcinoma (HGSC) is the most common histotype (70%) and is often diagnosed at stage III (51%) or IV (29%)^2,3^. Clear cell carcinoma (CCC) is a rare histotype (6%) in the United States and is often diagnosed at stage I (58%) or II (9%)^2^. However, CCC is more common in the East Asian population, where it accounts for 24% of all EOC^4,5^. HGSC is associated with *TP53* mutations and homologous recombination related genes mutations such as *BRCA1/2* and arises from fallopian tube^6^. Meanwhile, CCC is often associated with *ARID1A* and *PIK3CA* mutations and originates from endometriosis^7^. Clinical features of CCC are less sensitive to platinum based chemotherapy and CCC patients with advanced disease have a worse prognosis than that of HGSC^4,5,8–10^. EOC is a heterogeneous disease, we therefore may need to individualize treatment strategies according to each histotype^11^.

With the progress of immune-oncology, the immunological features of EOC is gradually becoming better understood. Suppression and activation of tumor-infiltrating T lymphocytes (TILs) are mediated by the interaction of various cytokines^12^. Host immune responses to tumor cells can be induced by tumor-infiltrating lymphocytes (TILs) present within the tumor microenvironment^13,14^. A recent large phase II study of pembrolizumab monotherapy in patients with advanced and recurrent EOC reported that while the overall response rate was 8%, there was a subgroup of cases showing a durable response in patients with EOC^15^. PD-L1 expression and the local immune-related gene expression profile were shown to be related to this response^16^. As such, most of the studies thus far have focused on the local tumor-immune microenvironment^13,14,17,18^. However, the systemic immunological background in patients with EOC has not been well studied.

Cytokines are a diverse group of proteins comprised of growth factors, interferons, and chemokines, which are involved in physiological activities and play an important role in cancer development or maintenance of the malignant phenotype^19–23^. Cytokines are secreted by numerous cell types, such as immune cells, endothelial cells, stromal cells and malignant cells. In general, a specific cytokine is secreted by more than one type of cells. Understanding their networks might unveil the systemic immune characteristics of EOC patients. Cytokine production and its control are highly complex and multifactorial, and their effects are reflected through multiple regulatory networks^24^. Therefore, evaluation of one single cytokine cannot address immunological networks of EOC as a whole system. To understand complex immunological networks in EOC, it is important to measure multiple serum soluble factors simultaneously and to evaluate them as a pattern of cytokine production, “cytokinome”, using so-called omics analysis. These comprehensive analyses of serum cytokines have not been performed in EOC to date.

In this study, we investigated a panel of cytokines in the sera taken before primary surgery from patients with EOC including HGSC, CCC and endometrioid carcinomas (EMC). This was done using multiplexed beads-based assays from which we subsequently analyzed the cytokinome in each histotype using cluster analysis to understand their clinical roles and immunological background in EOCs. We also identified subgroups characterized by specific cytokine profiles that had an impact on prognosis.

## Results

### Patient background and serum cytokines in patients with epithelial ovarian cancer

To investigate the clinical significance of the cytokine profile in patients with EOC, we examined a panel of serum cytokines including chemokines, interleukins and growth factors by multiplex bead-based immunoassay in the sera from 178 EOC patients. Table 1 shows the clinical background of 178 EOC patients included in this study. There were 73 HGSC, 66 CCC and 39 EMC cases. Patient background data stratified by histotype is shown in Supplementary Table 1A–C. Supplementary Table 2 shows the median and range of each value of serum cytokines. According to the distribution of the levels of the 27 cytokines in each patient, the raw data were transformed and normalized as described in the Materials and Methods section for further evaluation.

**Table 1:** Patient characteristics in all EOC. ^a^Peritoneal dissemination included dissemination limited to the pelvis. ^b^Evaluation of lymph node metastasis was performed by surgery or CT images.

Factors	
Number	178
Age (median [range])	57.5 [29.0, 84.0]
**Age category (%)**	
< 60	98 (55.1)
≥ 60	80 (44.9)
**Menopause (%)**	
No	56 (31.5)
Yes	122 (68.5)
**Primary site (%)**	
Ovary	165 (92.7)
Peritoneum	8 ( 4.5)
Tube	5 ( 2.8)
**Stage (%)**	
I	59 (33.1)
II	29 (16.3)
III	69 (38.8)
IV	21 (11.8)
**Histology (%)**	
Clear cell	66 (37.1)
Endometrioid	39 (21.9)
High-grade serous	73 (41.0)
**Ascites cytology (%)**	
Negative	66 (37.1)
Positive	112 (62.9)
**Peritoneal dissemination**^**a**^** (%)**	
No	81 (45.5)
Yes	97 (54.5)
**Lymph node metastasis**^**b**^** (%)**	
No	157 (88.2)
Yes	21 (11.8)
CEA (median [range])	1.3 [0.5, 337.2]
CA125 (median [range])	410.9 [7.7, 18,912.0]
CA19-9 (median [range])	19.0 [2.0, 23,926.6]

**Table 2:** Types of cytokines that correlated with histotype and its P value by multivariate analysis. ^a^*P* < 0.05.

Histotype	IL-1β	IL-5	IL-9	PDGF-BB	Eotaxin	IP-10	IL-1Ra	IL-2	IL-6	IL-10	MIP-1β	MCP-1
HGSC vs CCC	0.002^a^	< 0.001^a^	0.006^a^	< 0.001^a^	0.008^a^	< 0.001^a^	0.014^a^	0.943	0.005^a^	0.009^a^	0.007^a^	0.0025^a^
EMC vs CCC	0.007^a^	< 0.001^a^	0.007^a^	0.031^a^	0.016^a^	0.003^a^	0.586	0.031^a^	0.236	0.823	0.504	0.115

### Relationship between each cytokine and clinical features

Associations between each cytokine and clinicopathological factors of the patients were examined by multivariate regression analysis. Supplementary Table 3 describes the values of regression coefficient and *P*-values in the multivariate regression analysis for the associations among each cytokine and clinicopathological factors. As shown in Table 2, 12 of 27 cytokines (MIP-1β, IL-6, IL-1Ra, IL-5, IL-1β, Eotaxin, PDGF-bb, IP-10, MCP-1, IL-10, IL-9 and IL-2) were correlated with histotypes. In contrast, no correlations were observed between each cytokine and the International Federation of Gynecology and Obstetrics (FIGO) stage by multivariate analysis (Supplementary Table 3). Additionally, we analyzed the relationship between each cytokine and clinical features in each histotype in the same manner. There was no relationship between the cytokines and clinical features including stages in each histotype (Supplementary Table 4A–C).

**Table 3:** Cytokines included in each signature. ^a^Same cytokine between common signature of CCC and HGSC.

	Signature C1	Signature S1	Signature C2	Signature S2
Cytokines	IL-2^a^	IL-2^a^	IL-1β^a^	IL-1β^a^
	IL-6^a^	IL-6^a^	IL-4^a^	IL-4^a^
	IL-8^a^	IL-8^a^	IL-5^a^	IL-5^a^
	IL-15^a^	IL-15^a^	IL-7^a^	IL-7^a^
	VEGF^a^	VEGF^a^	IL-9^a^	IL-9^a^
	PDGF-BB^a^	PDGF-BB^a^	IL-10^a^	IL-10^a^
	GM-CSF^a^	GM-CSF^a^	IL-13^a^	IL-13^a^
	MIP-1β^a^	MIP-1β^a^	G-CSF^a^	G-CSF^a^
	Rantes^a^	Rantes^a^	TNF-α^a^	TNF-α^a^
	Eotaxin^a^	Eotaxin^a^	MIP-1α^a^	MIP-1α^a^
	IP-10^a^	IP-10^a^	IL-1Ra	IL-12(p70)
	MCP-1^a^	MCP-1^a^	IL-17	IFN-γ
	IL-12(p70)	IL-1Ra	bFGF	
	IFN-γ	IL-17		
		bFGF		

### Cluster analysis in each histotype

**Figure 1:**
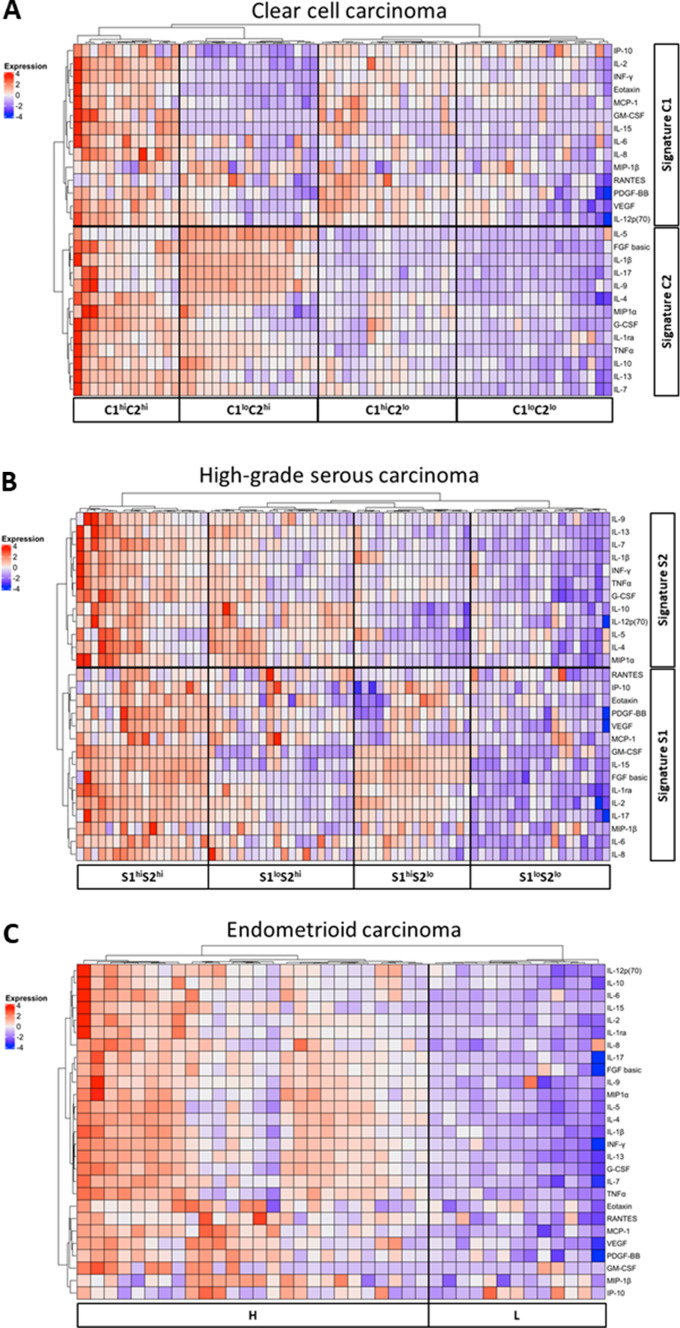
Heatmap of 27 serum cytokines in EOC patients. (**A**) Heatmap of 27 serum cytokines in 66 CCC patients. Hierarchical clustering showed 2 cytokine signatures (C1 and C2), which classified CCC patients into 4 clusters, namely as C1^hi^C2^hi^, C1^hi^C2^lo^, C1^lo^C2^hi^ and C1^lo^C2^lo^. (**B**) Heatmap of 27 serum cytokines in 73 HGSC patients. Hierarchical clustering also showed 2 cytokine signatures S1 and S2, and 4 clusters S1^hi^S2^hi^, S1^hi^S2^lo^, S1^lo^S2^hi^ and S1^lo^S2^lo^ for HGSC patients (**C**) Heatmap of 27 serum cytokines in 39 EMC patients. Hierarchical clustering did not show cytokine signatures, but EMC cases were categorized into two cytokine clusters, high or low overall cytokine production. R package pheatmap (version 1.0.12; https://www.rdocumentation.org/packages/pheatmap/versions/1.0.12) was used and freely available under a GPL-2 License.

We next performed cluster analysis in each histotype to identify subgroups based on the cytokine profile. As shown in Fig. 1A, cluster analysis of CCC revealed two distinct cytokine signatures, signature C1 and C2 (Table 3). Based on the high or low cytokine signature C1 and C2, CCC patients were categorized into 4 clusters, C1^hi^C2^hi^, C1^lo^C2^hi^, C1^hi^C2^lo^ and C1^lo^C2^lo^ (Fig. 1A). Similarly, cluster analysis for HGSC also classified them into 4 clusters based on the levels of cytokine signature S1 and S2 (Table 3 and Fig. 1B). Interestingly, signature S1 shared 12 of 15 (80%) cytokines (IL-2, IL-6, IL-8, IL-15, VEGF, GM-CSF, MIP1β, Rantes, Eotaxin, PDGF-BB, MCP-1, IP-10) with C1. Similarly, S2 shared 10 of 12 (83%) cytokines (IL-1β, IL-4, IL-5, IL-7, IL-9, IL-10, IL-13, G-CSF, TNF-α, MIP-1α) with C2. Most of the overlapping cytokines in C1 and S1 are involved in “chemotaxis and angiogenesis” from the point of view of their cytokine function. On the other hand, those in C2 and S2 are involved in “chronic inflammation” (Table 3). In contrast to CCC and HGSC patients, no obvious cytokine signature was found in EMC patients. However, EMC was categorized into 2 subgroups based on the high or low levels of overall cytokine production (Fig. 1C).

### Relationship between subgroups in each histotype and clinical outcomes

**Figure 2:**
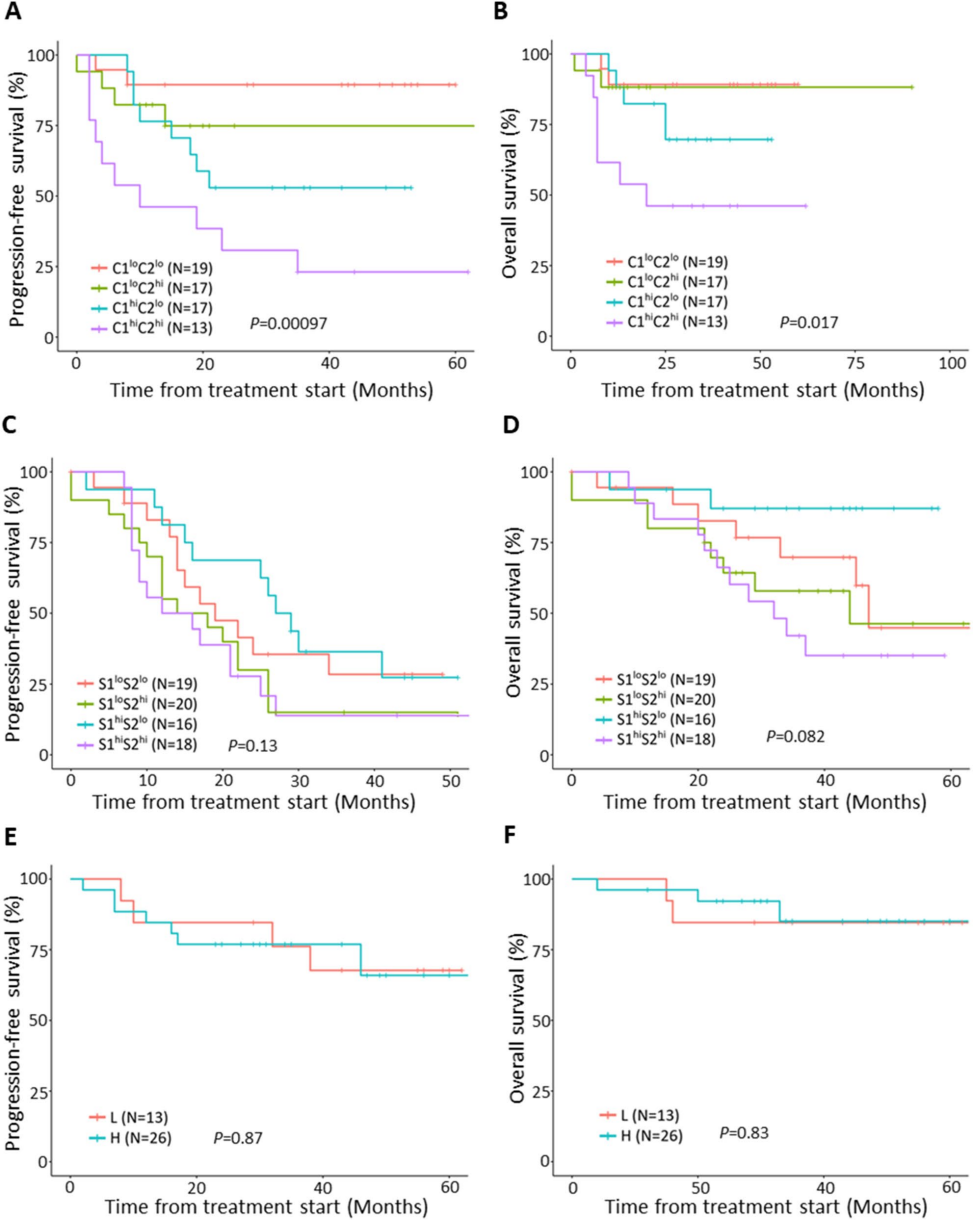
Clinical outcomes according to the cytokine clusters in each histotype. Kaplan–Meier curves for PFS (**A**) and OS (**B**) in 66 CCC patients stratified by the cytokine clusters based on C1 and C2 signatures. Kaplan–Meier curves for PFS (**C**) and OS (**D**) in 73 HGSC patients stratified by the cytokine clusters based on S1 and S2 signatures. Kaplan–Meier curves for PFS (**E**) and OS (**F**) in 39 EMC patients stratified by high (group H) or low (group L) overall cytokine production. No adjustment for multiplicity was made due to the exploratory nature of our analysis. R package survminer (version 0.4.6; https://www.rdocumentation.org/packages/survminer/versions/0.4.6) was used and freely available under a GPL-2 License.

Next, survival data stratified based on the cytokine clusters were analyzed in each histotype. As shown in Fig. 2A,B, cluster-based classification demonstrated significantly different progression-free and overall survivals (PFS and OS) in CCC patients (*P* = 0.00097 and *P* = 0.017). We found that patients with C1^hi^C2^hi^ cluster have the worst PFS and OS compared to other subgroups in CCC. The median PFS for patients with C1^hi^C2^hi^ and C1^hi^C2^lo^ were 10 and 20 months, respectively. HGSC did not have a significant difference with regard to PFS and OS (*P* = 0.13 and *P* = 0.082) based on the clustered cytokines (Fig. 2C,D). Notably, a high C1 signature was associated with worse survival in CCC patients (Supplementary Fig. 1A,B), but a high S1 signature alone was not associated with a worse prognosis in HGSC patients (Supplementary Fig. 1E,F), albeit C1 and S1 sharing 80% of cytokines. With regard to EMC, no correlation was found between the two clusters, high or low overall cytokines production, and clinical outcomes in patients in terms of PFS and OS (*P* = 0.87 and *P* = 0.83) (Fig. 2E,F).

Next, we performed cox proportional hazard regression analyses to assess the prognostic factors in patients with CCC. In univariate analysis, cytokine cluster was found to be prognostic factors for both PFS and OS (*P* = 0.0018 and *P* = 0.035, respectively) (Supplementary Table 5). In addition, a multivariate analysis was performed to examine the independent association between outcomes and cytokine clusters (Supplementary Table 5). After adjusting age and stage (I-II versus III-IV), cytokine cluster remained as an independent prognostic factor for PFS (*P* = 0.028), but it did not remain an independent prognostic factor for OS (*P* = 0.21) (Supplementary Table 5).

### Association between local cytokines gene expression and serum cytokines in CCC

**Figure 3:**
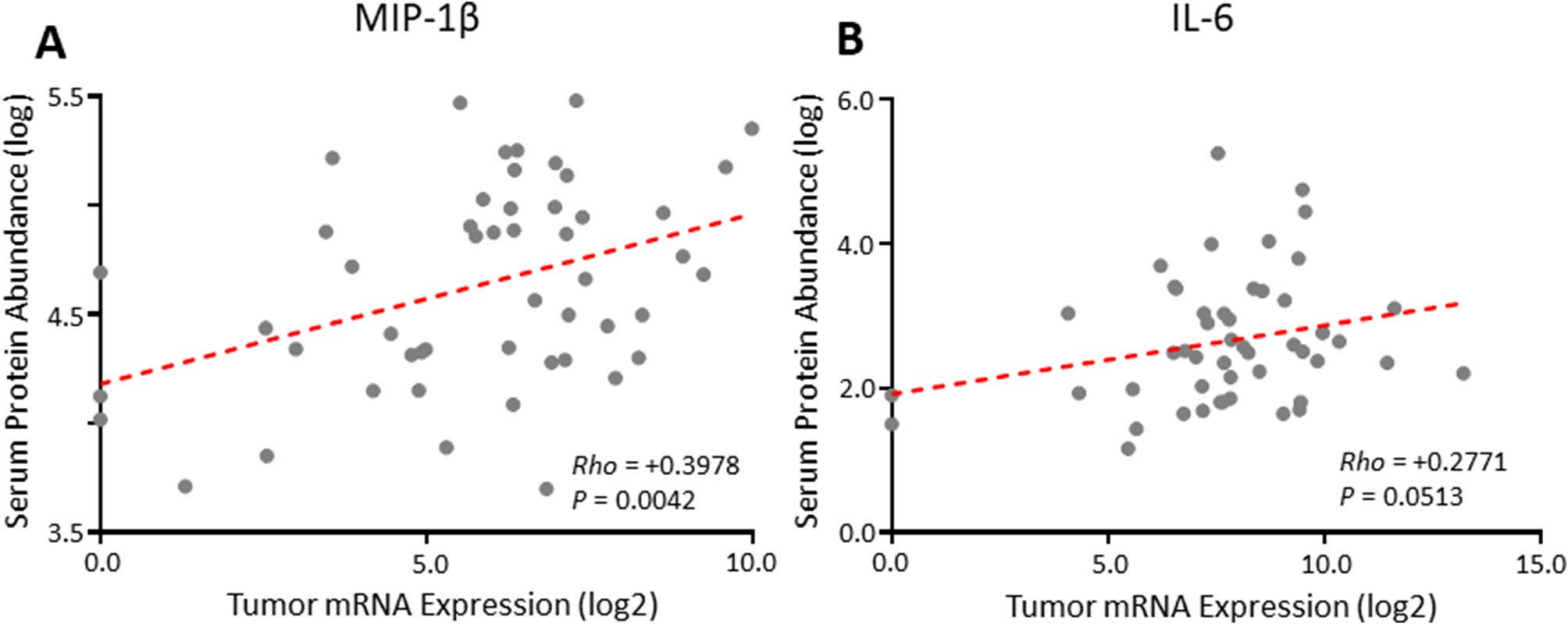
Correlation between local gene expression and serum cytokine for MIP-1β (**A**) and IL-6 (**B**). The strength of a linear relationship was calculated using the Pearson correlation coefficient. MATLAB (version 9.1.0.441655 (R2016b); https://www.mathworks.com/products/matlab.html?s_tid=hp_products_matlab) was used with a valid license.

Systemic and local cytokines may have different patterns in cancer patients. Thus, the local cytokine expression was investigated by digital multiplexed gene expression analysis for 51 cases of CCC available, and the association with those in the sera was analyzed for 26 cytokines that were included in both the serum cytokine array and immune related gene expression panel. As shown in Fig. 3A,B, and Supplementary Table 6, there was a correlation between serum cytokine and local cytokine gene expression for IL-6 and MIP-1β, but not for other 24 cytokines, suggesting a different cytokine environment in the blood versus the tumor in CCC patients.

## Discussion

In this study, we analyzed a panel of serum cytokines to understand the systemic immune background and its clinical role in EOC patients using multiplexed bead-based assays. Twelve of 27 cytokines correlated with histotypes of EOC. Cluster analyses identified 2 distinct cytokine profiles, which enabled categorization into a further 4 clusters in CCC and HGSC. These cytokine clusters are associated with different outcomes in CCC and HGSC.

The goal of cytokine studies in EOC thus far have focused on identifying diagnostic or prognostic markers^25–31^. Multiple cytokines were often measured in those studies, but analysis of serum cytokines was either performed individually or combined with just 2 or 3 cytokines after individual assessment^25,28,29,32^. Since systemic cytokine networks are complicated in cancer patients, an evaluation of the pattern of serum cytokines may be more appropriate rather than analyzing single individual cytokines. In this study we adopted a novel approach by evaluating a pattern of serum cytokines using the “omics” approach, often used in gene expression analysis, for each histotype of EOC patients.

Our exploratory analyses indicated that 12 of 27 cytokines correlated with histotypes. However, none of them correlated with FIGO stages. In contrast to most of the HGSC patients who had advanced stage tumors, CCC and EMC patients mostly had early stage tumors. As such, there is a possibility that this confounded our results. Serum IL-2, IL-6, IL-7, IL-8, IL-10, RANTES, MCP-1, MIP-1β, TNF-α, TNF-R2, VEGF, PIGF, PDGF-BB levels have been previously reported to be elevated in EOC patients^12,25,27,28,32–34^. An association between the levels of serum cytokines and FIGO stages was previously reported in EOC patients^28,32^, but histotypes were not considered in those studies, and no reports have addressed the relationship between serum cytokine profile and histotypes.

A cluster analysis for CCC demonstrated 2 cytokine signatures and 4 subgroups. We observed similar results in HGSC patients. Interestingly, each signature in CCC shared over 80% of cytokines with that in HGSC. The role of the overlapping cytokines in C1 and S1 seem to be involved in “chemotaxis and angiogenesis” and those of C2 and S2 in “chronic inflammation”, also known as “Th2-type cytokines”. These results might suggest that there could be a partly shared systemic immunological background in CCC and HGSC patients.

Four subgroups based on the clusters with high or low levels of each cytokine signature were associated with clinical outcomes in CCC but not for HGSC. Contrary to our expectations, different cytokine signatures may play a key role in determining outcomes in CCC and HGSC (Supplementary Fig. 1). Poorer outcomes were observed in patients with high levels of cytokines involved in chemotaxis and angiogenesis in CCC patients (Supplementary Fig. 1A,B). In contrast, these cytokines did not correlate with survival in HGSC patients (Supplementary Fig. 1E,F). Th2-type cytokines were associated with poorer outcomes in HGSC patients (Supplementary Fig. 1G,H). However, multiple testing was not considered for these analyses because of its exploratory nature. Our study demonstrated that the cytokine cluster based on C1 and C2 signatures was an independent prognostic factor for PFS by multivariate cox analysis in CCC patients. In contrast, we found cytokine cluster was a prognostic factor for OS in univariate analysis but not in multivariate analysis in this study. This might be partly due to the complex treatments for CCC patients after recurrence, or limited events for multivariate analysis on OS in this study. Unlike CCC, the outcomes of HGSC and EMC patients had no clear association with the levels of serum cytokines.

Only 2 out of 26 serum cytokines evaluated in this study were correlated with their local gene expression in CCC. This may be explained partly by the fact that cytokines in the blood are largely secreted by normal cells such as immune, endothelial or stromal cells rather than tumor cells.

Studying systemic immunological features is as important as studying the local tumor-immune microenvironment. Serum cytokine profiles could potentially serve as promising biomarkers for patient survival or aid in the selection of patients for various anti-cancer treatments, such as chemotherapies, targeted therapies and immunotherapies. Moreover, a longitudinal evaluation of serum cytokine signatures in EOC patients would enable us to understand the dynamic change in immunological features of patients during the course of treatment.

The limitations of the study include its retrospective design and inclusion of a relatively small number of each histotype of EOC patients. Also, these are initial exploratory analyses requiring studies in a larger cohort to confirm our findings. Despite these limitations, we identified several outcome-correlated cytokine clusters in CCC.

## Methods

### Patients and sera

The patients with EOC consisted of 392 consecutive cases that underwent staging laparotomy, exploratory laparotomy or debulking surgery at Saitama Medical University International Medical Center between January 2009 and September 2015. We included 178 EOC patients who were diagnosed as either HGSC, CCC or EMC and sera before surgery were available in this study (Supplementary Fig. 2). Patients’ sera were collected 1 day prior to the surgery and stored immediately at − 80 °C until use in the subsequent experiments. Relevant clinical and pathology data were respectively extracted from medical records and pathology reports. The clinical stage was classified according to the criteria of the International Federation of gynecology and Obstetrics (FIGO) staging system (1988). The histologic type of each tumor was reviewed by an expert pathologist in the field of gynecologic oncology. When the definitive pathological diagnosis was difficult morphologically, the final pathological diagnosis was performed using immunohistochemistry as an aid to the diagnosis.

### Serum cytokines measurement

Twenty-seven cytokines, including interleukin (IL)-1β, IL-1Ra, IL-2, IL-4, IL-5, IL-6, IL-7, IL-8, IL-9, IL-10, IL-12(p70), IL-13, IL15, IL-17, bFGF, Eotaxin, G-CSF, GM-CSF, IFN-γ, IP-10, MCP-1, MIP-1α, MIP-1β, PDGF-BB, RANTES, TNF-α, and VEGF in sera were measured using Bio-Plex Pro human Cytokine 27-plex Assay (Bio-Rad Laboratories, Inc., Hercules, CA, USA)^35^. Full details are described in Imai et al.^35^. Briefly, sera were incubated with microbeads labeled with specific antibodies to one of the aforementioned cytokines for 60 min. Following a washing step, the beads were incubated with the detection antibody cocktail with each antibody specific to a single cytokine for 30 min. After another washing step, the beads were incubated with streptavidin–phycoerythrin for 10 min, washed again, then the concentration of each cytokine was determined using the array reader.

### Cytokine gene expression analysis

RNA extraction was performed using QIAGEN RNeasy FFPE Kit from two slides of 5 µm-thick unstained formalin fixed paraffin embedded (FFPE) tumor specimens. In 66 cases of CCC whose serum cytokines were analyzed in this study, no left over or only a little remaining FFPE blocks were found in 10 of 66 cases. We excluded 5 out of 56 samples due to the poor quality, and performed the final analysis in 51 cases (Supplementary Fig. 2). The samples were subjected to gene expression profiling using the NanoString nCounter PanCancer Immune Profiling Panel codesets and IO360 codesets (NanoString Technologies Inc; Seattle, WA) according to the manufacturer’s instructions. The gene expression data normalization was performed using nSolver analysis software version 3.0 (NanoString Technologies Inc; Seattle, WA). The raw count from NanoString was subjected to background subtraction, positive control normalization and housekeeping genes normalization as defined by the PanCancer Immune Profiling Panel. Local cytokines gene expression was extracted from the normalized data.

### Data analysis and statistics

To investigate associations between serum soluble factors and patient characteristics, we used multivariate linear regression models. All cytokines/chemokines/growth factors were log(X + 1) transformed and normalized. Each serum soluble factor was defined as a dependent variable, and a panel of patient characteristics were set as independent variables. Serum CEA, CA125 and CA19-9 were log(log(X + 1)) transformed for multivariate analysis. The natural logarithm was used in all data values of logarithm. *P*-values for those associations were calculated, and *P* < 0.05 was considered statistically significant. Because the analysis was exploratory, multiple testing was not considered. All statistics were performed using R 3.4.2 (R Foundation for Statistical Computing). Pearson correlation coefficient test was conducted using MATLAB version 9.1.0.441655 (R2016b), and statistics and machine learning toolbox version 11.0 (MathWorks; Natick, MA).

### Cluster and survival analysis

Cluster analysis was performed for all soluble factors for EOC and each histotype using ‘manhattan’ distance measure and ‘ward.D’ agglomeration method of R package pheatmap (version 1.0.8). We identified subgroups of EOC based on the finding of cluster analysis. The R package survminer (version 0.4.6) was used for survival analysis. The Kaplan–Meier method was performed to construct survival curves, and the log-rank test determined the significance between these subgroups. Cox proportional hazard regression model was also used to perform univariate and multivariate survival analyses. OS was defined as the time from primary surgery to the date of death. PFS was defined as the time from primary surgery to the date of occurrence of an event (death, progression or relapse of EOC). Computed tomography (CT) scan was used to assess progression events for all patients and imaging assessment was performed according to response evaluation criteria in solid tumours: RECIST (version 1.1) criteria.

### Ethics approval

The study was conducted under review board at Saitama Medical University International Medical Center (no.13-092). The procedures used in this study adhere to the tenets of the Declaration of Helsinki.

### Consent to participate

Informed consents including future research purposes were obtained from all patients in previous studies (no. 10-078 and 12-096), and the Institutional Review Board approved to use the research materials in the current study.

### Consent for publication

Not applicable.

## Supplementary information

Supplementary Information

## Data Availability

All data generated or analyzed during this study are included in this published article and its supplementary information files.
