# Supplementary material for: Identification of serum cytokine clusters associated with outcomes in ovarian clear cell carcinoma
Source: Sci Rep. 2020 Oct 28;10:18503. doi: 10.1038/s41598-020-75536-1 (PMC7595156; doi:10.1038/s41598-020-75536-1)
Supplement: Supplementary file 1 — Supplementary Information [file 41598_2020_75536_MOESM1_ESM.pdf]

Supplementary Information

Identification of serum cytokine clusters associated with outcomes in ovarian clear cell carcinoma

Akira Yabuno, Hirokazu Matsushita[*#+], Tetsutaro Hamano, Tuan Zea Tan, Daisuke Shintani, Nao Fujieda, David SP Tan, Ruby Yun-Ju Huang, Keiichi Fujiwara, Kazuhiro Kakimi and Kosei Hasegawa[*#]

[#] Senior authors

[+] Current address: Aichi Cancer Center Research Institute, Aichi, Japan

[*] Corresponding authors

[*] Correspondence to: koseih@saitama-med.ac.jp (KH), and h.matsushita@aichi-cc.jp (HM)

Supplementary Figures

Supplementary Figure 1

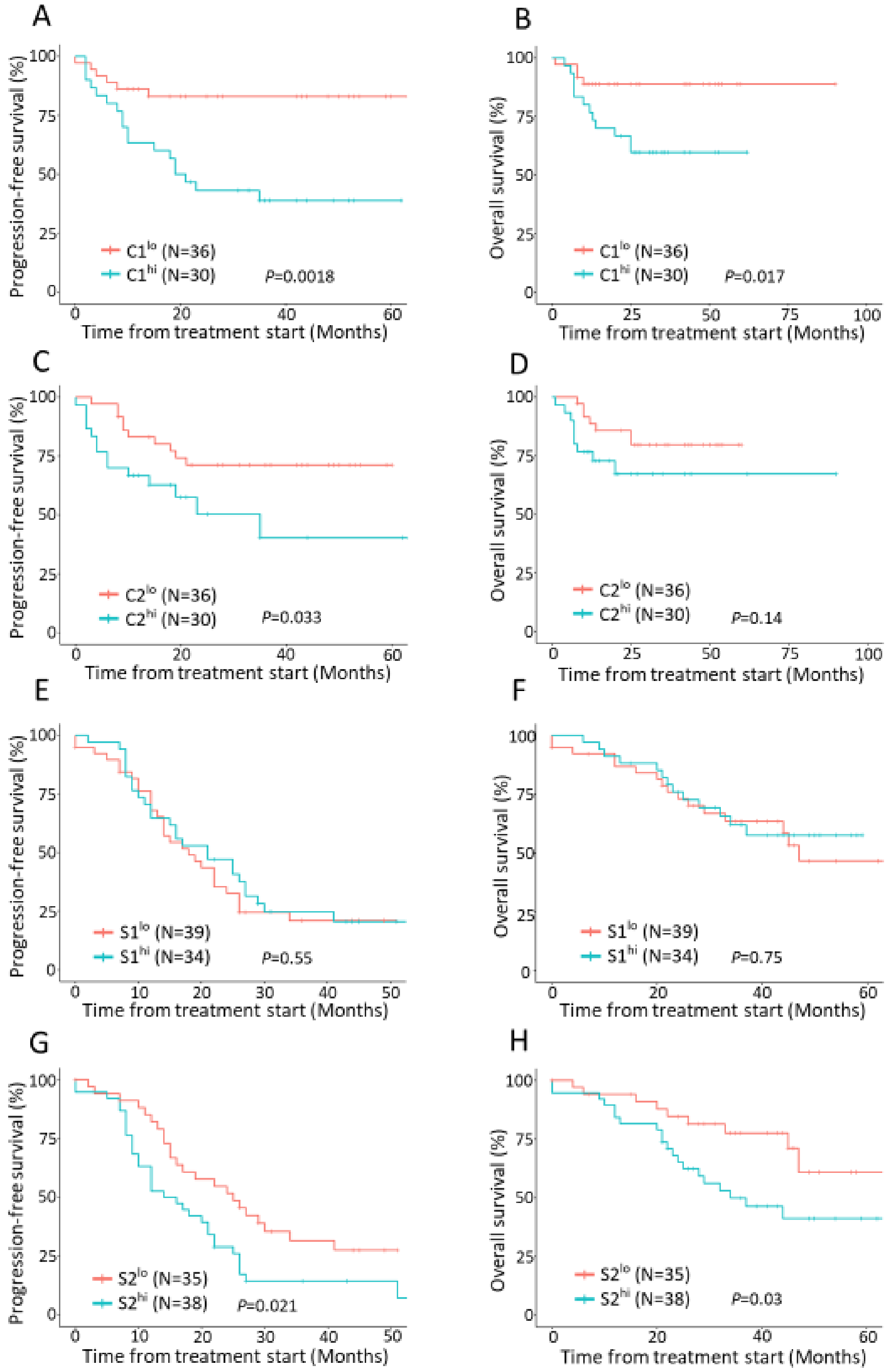

Figure S1. Clinical outcomes according to the cytokine signatures in CCC and HGSC. Kaplan-Meier curves for PFS (A) and OS (B) in CCC patients based on high or low C1 signature ($C1^{hi}C2^{hi}$+$C1^{hi}C2^{lo}$ vs. $C1^{lo}C2^{hi}$+$C1^{lo}C2^{lo}$). Kaplan-Meier curves for PFS (C) and OS (D) in CCC patients based on high or low C2 signature ($C1^{hi}C2^{hi}$+$C1^{lo}C2^{hi}$ vs. $C1^{hi}C2^{lo}$+$C1^{lo}C2^{lo}$). Kaplan-Meier curves for PFS (E) and OS (F) in HGSC patients based on high or low S1 signature ($S1^{hi}S2^{hi}$+$S1^{hi}S2^{lo}$ vs. $S1^{lo}S2^{hi}$+$S1^{lo}S2^{lo}$). Kaplan-Meier curves for PFS (G) and OS (H) in HGSC patients based on high or low S2 signature ($S1^{hi}S2^{hi}$+$S1^{lo}S2^{hi}$ vs. $S1^{hi}S2^{lo}$+$S1^{lo}S2^{lo}$).

Supplementary Figure 2

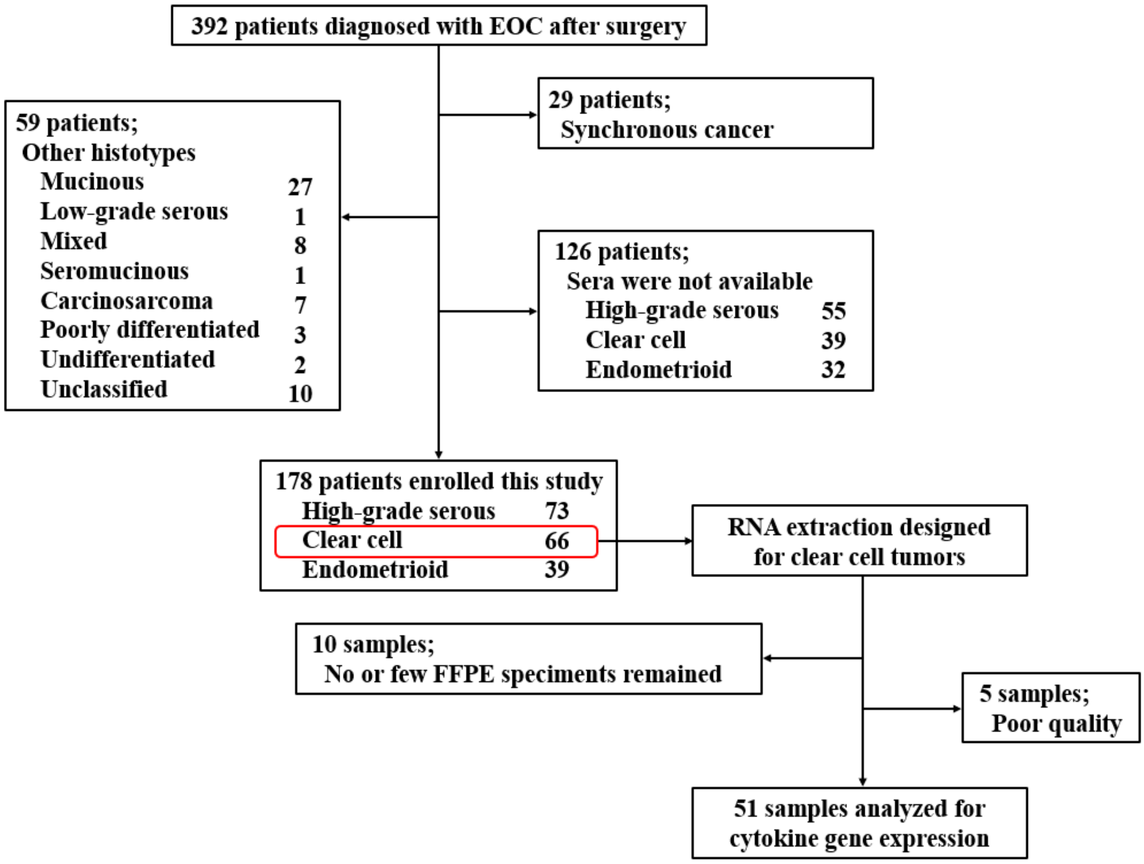

Figure S2. Flowchart of study identification and inclusion. FFPE, formalin fixed paraffin embedded

Supplementary Tables

Supplementary Table 1A. Patient background by each histologic type

	level	Clear cell	Endometrioid	High-grade serous	p
patients number		66	39	73	
Age (median [range])		54.5 [37.0, 80.0]	57.0 [29.0, 80.0]	61.0 [33.0, 84.0]	0.003
Age_category (%)	< 60	44 (66.7)	22 (56.4)	32 (43.8)	0.025
	≥ 60	22 (33.3)	17 (43.6)	41 (56.2)	
Menopause (%)	No	27 (40.9)	13 (33.3)	16 (21.9)	0.053
	Yes	39 (59.1)	26 (66.7)	57 (78.1)	
Primary_site (%)	Ovary	66 (100.0)	39 (100.0)	60 (82.2)	< 0.001
	Peritoneum	0 (0.0)	0 (0.0)	8 (11.0)	
	Tube	0 (0.0)	0 (0.0)	5 (6.8)	
Stage (%)	I, II	52 (78.8)	30 (76.9)	6 (8.2)	< 0.001
	III, IV	14 (21.2)	9 (23.1)	67 (91.8)	
Histology (%)	Clear cell	66 (100.0)	0 (0.0)	0 (0.0)	< 0.001
	Endometrioid	0 (0.0)	39 (100.0)	0 (0.0)	
	Serous	0 (0.0)	0 (0.0)	73 (100.0)	
Ascites_cytology (%)	Negative	35 (53.0)	22 (56.4)	9 (12.3)	< 0.001
	Positive	31 (47.0)	17 (43.6)	64 (87.7)	
Peritoneal dissemination (%)	No	52 (78.8)	25 (64.1)	4 (5.5)	< 0.001
	Yes	14 (21.2)	14 (35.9)	69 (94.5)	
Lymph node metastasis (%)	No	63 (95.5)	36 (92.3)	58 (79.5)	0.009
	Yes	3 (4.5)	3 (7.7)	15 (20.5)	

Supplementary Table 1B. Patient background by each histologic type

	level	Clear cell	Endometrioid	High-grade serous	p
CEA (median [range])		1.2 [0.5, 26.1]	2.8 [0.5, 337.2]	1.1 [0.5, 146.6]	< 0.001
CA125 (median [range])		121.7 [10.4, 5255.7]	181.1 [7.7, 4340.8]	977.1 [26.8, 18912.0]	< 0.001
CA19-9 (median [range])		40.9 [2.0, 23926.6]	58.8 [2.0, 8580.1]	6.8 [2.0, 1335.6]	< 0.001
Tumor size (mean (sd))		132.0 (47.7)	114.6 (45.3)	80.3 (38.2)	< 0.001
Primary surgery (%)	No	19 (28.8)	8 (20.5)	52 (71.2)	< 0.001
	Yes	47 (71.2)	31 (79.5)	21 (28.8)	
Residual tumor (%)	No	52 (78.8)	28 (71.8)	12 (16.4)	< 0.001
	Yes	14 (21.2)	11 (28.2)	61 (83.6)	
PFS event (%)	No	41 (62.1)	28 (71.8)	17 (23.3)	< 0.001
	Yes	25 (37.9)	11 (28.2)	56 (76.7)	
OS event (%)	No	50 (75.8)	34 (87.2)	44 (60.3)	0.007
	Yes	16 (24.2)	5 (12.8)	29 (39.7)	
TFI (median [range])		18.5 [0.0, 60.0]	26.0 [0.0, 62.0]	11.0 [0.0, 49.0]	< 0.001
Platinum sencitivity (%)	Partially sensitive	9 (13.6)	3 (7.7)	15 (20.5)	0.039
	Resistant	15 (22.7)	5 (12.8)	22 (30.1)	
	Sensitive	42 (63.6)	31 (79.5)	36 (49.3)	
PFS (median [range])		21.0 [0.0, 63.0]	32.0 [2.0, 66.0]	18.0 [0.0, 54.0]	< 0.001
OS (median [range])		25.5 [1.0, 90.0]	35.0 [4.0, 66.0]	34.0 [0.0, 65.0]	0.006

Supplementary Table 1C. Patient background by each histologic type

level	Clear cell	Endometrioid	High-grade serous	p
MIP1β (median [range])	105.8 [39.4, 238.8]	117.1 [38.3, 259.7]	140.4 [67.8, 661.2]	< 0.001
IL6 (median [range])	10.1 [2.2, 189.6]	8.5 [3.5, 120.6]	13.3 [3.4, 45.3]	0.09
IFNγ (median [range])	214.2 [61.1, 27861.0]	321 [46.0, 819.6]	287.2 [99.3, 7246.7]	0.004
IL1Ra (median [range])	107.8 [47.5, 17167.5]	103.9 [17.8, 5526.4]	125.7 [34.4, 431.1]	0.546
IL5 (median [range])	6.4 [2.2, 81.9]	4.6 [0.8, 15.9]	4.4 [0.9, 16.4]	< 0.001
GMCSF (median [range])	0.0 [0.0, 1118.3]	0.0 [0.0, 135.9]	24.6 [0.0, 281.8]	< 0.001
TNFα (median [range])	98.3[42.1, 5984.4]	97.4 [40.9, 243.5]	101.9[37.1, 1164.1]	0.488
Rantes (median [range])	19051.5 [2719.0, 127126.2]	16721.6 [3779.4, 76096.2]	14948.6 [5594.6, 231100.8]	0.07
IL2 (median [range])	3.5 [0.0, 119.3]	5.3 [0.0, 425.6]	6.8 [0.5, 20.7]	< 0.001
IL1β (median [range])	2.7 [0.8, 163.3]	2.3 [0.5, 6.8]	2.6 [1.0, 37.7]	0.251
Eotaxin (median [range])	214.6 [43.2, 16730.0]	288.8 [110.4, 632.5]	385.6 [116.8, 973.7]	< 0.001
bFGF (median [range])	38.7 [16.8, 186.2]	34.7 [0.0, 146.9]	45.6 [13.6, 280.6]	0.026
VEGF (median [range])	100.6 [4.6, 1187.4]	94.2 [3.7, 1031.3]	221.8 [1.8, 2522.5]	< 0.001
PDGFBB (median [range])	5066.4 [270.7, 16834.7]	6233.6 [336.1, 19698.9]	8673.8 [530.0, 91628.8]	< 0.001
IP10 (median [range])	1989.2 [355.4, 11455.3]	2920.7 [1389.2, 16499.6]	7977.5 [682.8, 84449.3]	< 0.001
IL13 (median [range])	12.6 [3.1, 228.4]	14.3 [1.7, 46.3]	12.6 [5.5, 93.1]	0.793
IL4 (median [range])	9.3 [2.5, 28.6]	9.1 [3.6, 22.2]	8.6 [4.4, 22.5]	0.688
MCP1 (median [range])	25.0 [6.9, 395.5]	29.6 [6.9, 123.4]	33.5 [10.7, 328.2]	0.002
IL8 (median [range])	18.2 [8.3, 128.6]	22.1 [8.8, 73.0]	27.4 [11.3, 230.2]	< 0.001
MIP1α (median [range])	3.8 [1.5, 14.3]	4.2 [1.8, 12.0]	4.2 [1.9, 10.3]	0.614
IL10 (median [range])	15.8 [4.0, 377.0]	14.0 [0.2, 1466.3]	13.0 [2.1, 93.8]	0.231
GCSF (median [range])	57.7 [30.5, 426.2]	61.6 [18.6, 187.8]	72.1 [30.5, 198.9]	0.016
IL15 (median [range])	0.0 [0.0, 70.1]	0.6 [0.0, 225.4]	3.8 [0.0, 12.5]	< 0.001
IL7 (median [range])	22.5 [5.9, 398.8]	24.1 [3.1, 74.0]	24.6 [10.4, 153.2]	0.276
IL12(p70) (median [range])	84.9 [1.2, 1381.0]	85.9 [12.2, 2577.5]	119.4 [9.0, 1015.7]	0.2
IL17a (median [range])	65.1 [11.3, 859.0]	65.1 [0.0, 553.9]	68.8 [7.2, 932.5]	0.428
IL9 (median [range])	39.1 [13.4, 1326.3]	39.7 [9.1, 214.8]	37.5 [16.9, 498.8]	0.34

Supplementary Table 2. Median and range of serum cytokines in all EOC patients

Cytokines	median [range]
MIP1β	122.7 [38.3, 661.2]
IL6	11.0 [2.2, 189.6]
IFNg	285.7 [46.0, 27861.0]
IL1Ra	121.1 [17.8, 17167.5]
IL5	4.9 [0.8, 81.9]
GMCSF	0.0 [0.0, 1118.3]
TNFα	101.5 [37.1, 5984.4]
Rantes	16217.9 [2719.0, 231100.8]
IL2	5.0 [0.0, 425.6]
IL1β	2.4 [0.5, 163.3]
Eotaxin	277.6 [43.2, 16730.0]
bFGF	41.5 [0.0, 280.6]
VEGF	136.2 [1.8, 2522.5]
PDGFBB	6295.8 [270.7, 91628.8]
IP10	3781.6 [355.4, 84449.3]
IL13	12.6 [1.7, 228.4]
IL4	9.1 [2.5, 28.6]
MCP1	27.7 [6.9, 395.5]
IL8	23.2 [8.3, 230.2]
MIP1α	4.0 [1.5, 14.3]
IL10	14.2 [0.2, 1466.3]
GCSF	64.7 [18.6, 426.2]
IL15	1.3 [0.0, 225.4]
IL7	23.9 [3.1, 398.8]
IL12(p70)	99.5 [1.2, 2577.5]
IL17	67.1 [0.0, 932.5]
IL9	38.5 [9.1, 1326.3]

Supplementary Table 3. Associations between cytokines and patient characteristics

	Cytokines/chemokines (estimates and p-values)																										
	MIP-1β	IL-6	IFN-γ	IL-1Ra	IL-5	GM-CSF	TNF-α	Rantes	IL-2	IL-1β	Eotaxin	bFGF	VEGF	PDGF-BB	IP-10	IL-13	IL-4	MCP-1	IL-8	MIP-1α	IL-10	G-CSF	IL-15	IL-7	IL-12(p70)	IL-17	IL-9
Age	0.003	0.001	-0.008	-0.009	-0.006	-0.033	-0.009	-0.005	-0.003	-0.01	0.005	-0.002	0.009	-0.01	0.006	-0.006	-0.004	-0.004	-0.001	-0.003	0	-0.004	-0.011	-0.007	0.001	-0.006	-0.005
	0.511	0.915	0.26	0.242	0.429	0.079	0.144	0.449	0.685	0.074	0.443	0.692	0.447	0.259	0.477	0.298	0.328	0.576	0.783	0.399	0.959	0.404	0.26	0.182	0.928	0.454	0.472
Menopause = Yes	0.057	0.054	0.028	0.21	0.234	1.172*	0.199	0.109	-0.097	0.287*	-0.069	0.143	-0.053	0.152	0.027	0.121	0.06	0.036	0.109	0.007	-0.031	0.021	0.339	0.198	0.048	0.221	0.196
	0.644	0.757	0.874	0.26	0.25	0.012	0.167	0.524	0.646	0.033	0.676	0.348	0.852	0.486	0.904	0.394	0.53	0.831	0.384	0.925	0.882	0.853	0.145	0.15	0.832	0.284	0.282
Stage = III, IV	0.256	0.173	-0.081	0.138	0.414	-0.262	0.041	-0.202	-0.083	0.229	0.061	-0.101	0.036	0.202	-0.183	0.093	0.108	0.081	0.125	-0.109	0.206	-0.028	0.044	0.123	0.185	-0.036	0.251
	0.102	0.433	0.718	0.559	0.108	0.65	0.822	0.353	0.757	0.177	0.769	0.601	0.92	0.464	0.511	0.606	0.375	0.705	0.427	0.263	0.431	0.846	0.881	0.479	0.517	0.891	0.275
Histology = Endometrioid	0.068	-0.171	0.156	-0.084	-0.685*	0.022	-0.148	-0.128	0.377*	-0.304*	0.332*	-0.206	0.201	0.390*	0.568*	-0.038	0.009	0.221	0.105	0.062	-0.038	0.066	0.197	-0.081	0.18	-0.212	-0.412*
	0.504	0.236	0.283	0.586	<0.001	0.955	0.21	0.368	0.031	0.007	0.016	0.102	0.389	0.031	0.003	0.746	0.905	0.115	0.31	0.33	0.823	0.489	0.303	0.474	0.335	0.213	0.007
Histology = High-grade serous	0.322*	-0.473*	0.106	-0.436*	-0.991*	0.531	-0.245	0.013	-0.014	-0.411*	0.415*	-0.13	0.348	0.859*	0.981*	-0.174	-0.143	0.360*	0.009	-0.015	-0.516*	-0.068	0.236	-0.085	-0.107	-0.206	-0.477*
	0.007	0.005	0.522	0.014	<0.001	0.218	0.071	0.934	0.943	0.002	0.008	0.365	0.193	<0.001	<0.001	0.194	0.115	0.025	0.937	0.831	0.009	0.528	0.281	0.512	0.616	0.288	0.006
Ascites cytology = Positive	0.067	0.07	0.151	0.006	-0.083	0.058	0.061	0.008	0.12	0.025	0.04	0.007	-0.291	-0.207	-0.185	0.026	0.01	-0.012	0.033	0.11	-0.166	0.111	0.113	-0.017	-0.16	-0.005	-0.059
	0.47	0.593	0.254	0.967	0.585	0.865	0.569	0.954	0.447	0.801	0.744	0.95	0.17	0.205	0.261	0.809	0.889	0.922	0.723	0.057	0.284	0.201	0.515	0.872	0.345	0.975	0.663
Peritoneal dissemination = Yes	-0.176	0.176	0.168	0.105	-0.284	0.978	0.111	-0.247	0.405	-0.101	0.09	0.14	0.039	-0.467	0.614*	0.075	-0.031	-0.036	0.248	0.094	-0.046	0.156	0.263	-0.016	-0.007	0.041	0.059
	0.229	0.396	0.422	0.635	0.238	0.072	0.512	0.225	0.106	0.522	0.644	0.438	0.909	0.071	0.02	0.654	0.786	0.859	0.095	0.303	0.852	0.255	0.339	0.922	0.978	0.867	0.785
Lymph node metastasis = Yes	-0.098	0.001	-0.031	-0.057	-0.026	0.162	-0.026	0.08	0.074	-0.007	-0.219	0.133	0.179	0.163	-0.136	-0.032	-0.015	-0.12	-0.058	0.097	-0.047	0.055	-0.015	0.006	0.059	0.211	0.016
	0.409	0.997	0.854	0.751	0.895	0.712	0.848	0.63	0.713	0.954	0.167	0.362	0.511	0.436	0.52	0.813	0.87	0.46	0.628	0.19	0.815	0.619	0.946	0.966	0.787	0.287	0.927
CEA	0.044	-0.176	-0.01	-0.047	0.067	-0.302	-0.004	-0.188	-0.065	0.01	-0.018	-0.154	-0.291	-0.290*	-0.107	-0.019	-0.029	-0.055	-0.054	-0.044	-0.139	-0.036	-0.072	-0.078	-0.198	-0.194	-0.026
	0.542	0.083	0.919	0.664	0.569	0.256	0.959	0.06	0.593	0.896	0.85	0.082	0.078	0.023	0.403	0.821	0.605	0.577	0.452	0.321	0.247	0.596	0.596	0.326	0.132	0.106	0.802
CA125	0.073	0.690*	0.149	0.485*	0.552*	0.064	0.287	0.248	0.227	0.370*	0.005	0.372*	0.798*	0.219	0.422	0.340*	0.182	0.104	0.321*	0.044	0.684*	0.194	0.341	0.343*	0.717*	0.328	0.19
	0.616	0.002	0.477	0.03	0.023	0.906	0.093	0.224	0.363	0.021	0.981	0.04	0.019	0.395	0.107	0.045	0.11	0.605	0.031	0.63	0.006	0.156	0.216	0.036	0.008	0.18	0.377
CA19-9	0.041	0.05	-0.037	-0.164	0.062	-0.191	-0.041	-0.008	-0.187	-0.015	-0.123	-0.071	-0.005	-0.055	-0.167	0.018	0.012	0.019	0.116	0.034	-0.028	-0.032	-0.219	-0.013	-0.028	-0.014	0.116
	0.548	0.607	0.71	0.118	0.582	0.454	0.605	0.93	0.114	0.842	0.182	0.4	0.974	0.65	0.175	0.818	0.818	0.845	0.098	0.424	0.806	0.616	0.092	0.862	0.828	0.906	0.253
Constant	4.212*	1.234*	5.652*	4.593*	1.752*	1.961	4.526*	9.786*	1.523*	1.274*	5.145*	3.305*	3.026*	8.641*	6.688*	2.261*	2.196*	3.293*	2.333*	1.579*	1.853*	3.998*	0.582	2.913*	3.276*	3.952*	3.701*
	<0.001	0.006	<0.001	<0.001	0.001	0.089	<0.001	<0.001	0.005	0.001	<0.001	<0.001	<0.001	<0.001	<0.001	<0.001	<0.001	<0.001	<0.001	<0.001	0.001	<0.001	0.319	<0.001	<0.001	<0.001	<0.001
Observations	178	178	178	178	178	178	178	178	178	178	178	178	178	178	178	178	178	178	178	178	178	178	178	178	178	178	178
Adjusted R^2	0.146	0.14	0.005	0.019	0.184	0.125	0.006	0.028	0.075	0.073	0.128	0.049	0.078	0.144	0.401	0.009	-0.014	0.018	0.2	0.008	0.027	0.038	0.132	0.021	0.032	-0.008	0.037
F Statistic (df = 11; 166)	3.759*	3.623*	1.078	1.307	4.625*	3.300*	1.089	1.456	2.305*	2.265*	3.363*	1.83	2.369*	3.713*	11.765*	1.14	0.771	1.294	5.022*	1.131	1.454	1.628	3.456*	1.341	1.536	0.877	1.617

The first row is coefficient and the second row is p-value for each parameter.
All cytokines/chemokines were log(x+1) transformed. CEA, CA125 and CA19-9 were log(log(x+1)) transformed.
No adjustment for multiplicity was made due to the exploratory nature of our analysis.
*p<0.05

Supplementary Table 4A. Associations between cytokines and patient characteristics in HGSC

	Cytokines/chemokines (coefficient and p-values)																										
	MIP-1β	IL-6	IFN-γ	IL-1Ra	IL-5	GM-CSF	TNF-α	Rantes	IL-2	IL-1β	Eotaxin	bFGF	VEGF	PDGF-BB	IP-10	IL-13	IL-4	MCP-1	IL-8	MIP-1α	IL-10	G-CSF	IL-15	IL-7	IL-12(p70)	IL-17	IL-9
Age	0.003	-0.002	-0.005	-0.009	0.003	-0.043	-0.004	-0.016	-0.007	-0.006	0.004	-0.009	-0.006	-0.014	-0.001	-0.007	0.002	-0.003	0.002	0	-0.002	-0.005	-0.023	-0.007	-0.005	-0.005	-0.004
	0.656	0.839	0.526	0.238	0.641	0.119	0.541	0.068	0.368	0.29	0.604	0.256	0.675	0.267	0.933	0.325	0.621	0.735	0.757	0.906	0.838	0.369	0.063	0.289	0.664	0.578	0.62
Menopause = Yes	0.195	0.201	0.026	0.188	-0.2	1.611*	0.079	0.164	0.164	0.117	0.015	0.296	0.357	0.621	0.254	0.136	-0.046	0.018	0.189	-0.135	-0.118	0.053	0.916*	0.213	0.104	0.161	0.076
	0.334	0.402	0.908	0.375	0.303	0.036	0.679	0.481	0.462	0.474	0.944	0.178	0.398	0.069	0.481	0.469	0.697	0.949	0.367	0.216	0.653	0.731	0.009	0.263	0.73	0.525	0.739
Stage = III, IV	0.373	0.855	0.442	0.323	0.476	-0.487	0.078	0.437	0.118	0.216	0.256	-0.052	0.705	1.057	0.354	0.5	0.450*	0.889	0.604	0.228	0.795	0.362	0.733	0.521	0.598	0.276	0.494
	0.305	0.051	0.28	0.397	0.175	0.721	0.821	0.3	0.769	0.462	0.498	0.895	0.356	0.085	0.586	0.144	0.04	0.077	0.112	0.246	0.096	0.196	0.232	0.132	0.275	0.547	0.23
Ascites cytology = Positive	0.087	-0.043	-0.003	0.054	0.121	-0.288	0.012	-0.15	0.022	0.126	-0.032	-0.076	-0.657	-0.697*	0	0.082	-0.035	-0.087	-0.087	0.175	0.035	0.05	-0.242	0.031	-0.155	-0.019	0.045
	0.641	0.847	0.991	0.786	0.505	0.683	0.945	0.49	0.918	0.407	0.868	0.709	0.099	0.03	0.004	0.641	0.755	0.734	0.655	0.087	0.886	0.73	0.445	0.86	0.582	0.937	0.831
Peritoneal dissemination = Yes	-0.478	-0.951	-0.879	-0.296	-0.533	0.556	-0.383	-0.887	0.128	-0.586	-0.051	0.479	-0.32	-1.254	0.138	-0.5	-0.335	-1.01	-0.339	-0.25	-0.681	-0.26	-0.711	-0.7	-0.286	-0.011	-0.17
	0.275	0.071	0.077	0.518	0.206	0.734	0.353	0.082	0.791	0.1	0.911	0.313	0.727	0.089	0.86	0.223	0.198	0.094	0.454	0.289	0.233	0.439	0.334	0.093	0.663	0.984	0.729
Lymph node metastasis = Yes	-0.108	-0.013	-0.142	-0.096	-0.08	0.202	-0.111	0.06	-0.001	-0.048	-0.234	-0.047	0.289	0.11	-0.01	-0.048	-0.108	-0.103	-0.146	-0.031	0.046	-0.091	0.127	-0.021	0.107	-0.048	-0.13
	0.46	0.939	0.388	0.532	0.572	0.713	0.422	0.724	0.993	0.682	0.127	0.768	0.347	0.653	0.968	0.725	0.216	0.607	0.337	0.691	0.809	0.42	0.607	0.881	0.628	0.794	0.431
CEA	0.01	-0.147	0.088	-0.022	0.097	-0.33	0.106	-0.205	0.077	0.061	-0.08	0.029	-0.428	-0.383	-0.308	-0.027	0.048	-0.306	-0.063	0.033	-0.17	0.068	-0.224	-0.069	-0.233	-0.006	0.011
	0.932	0.286	0.497	0.854	0.384	0.448	0.331	0.129	0.548	0.512	0.504	0.816	0.081	0.051	0.139	0.806	0.479	0.056	0.599	0.595	0.259	0.445	0.25	0.528	0.182	0.97	0.934
CA125	-0.185	-0.105	0.343	0.252	0.501	-1.456	0.237	0.468	0.021	0.193	-0.201	-0.183	-0.384	-0.056	0.547	0.092	0.313	0.021	0.235	0.195	0.285	0.224	-0.35	0.089	0.139	-0.097	-0.07
	0.545	0.774	0.32	0.432	0.092	0.207	0.412	0.188	0.951	0.436	0.526	0.582	0.55	0.913	0.319	0.749	0.087	0.961	0.459	0.239	0.475	0.342	0.497	0.757	0.763	0.801	0.839
CA19-9	0.071	-0.075	0.236	0.001	0.283*	-0.184	0.105	0.123	-0.046	0.081	-0.077	-0.148	0.045	-0.073	-0.01	0.246*	0.166*	0.263	0.109	0.143*	0.31	0.043	-0.276	0.122	0.16	-0.023	0.103
	0.587	0.634	0.113	0.994	0.028	0.71	0.397	0.419	0.753	0.445	0.574	0.303	0.87	0.739	0.965	0.049	0.036	0.147	0.423	0.048	0.074	0.673	0.215	0.328	0.418	0.892	0.489
Constant	5.054*	2.970*	5.694*	4.689*	0.45	6.599*	4.616*	10.018*	2.041*	1.453*	5.931*	4.374*	6.190*	10.276*	8.200*	2.543*	1.455*	3.786*	2.458*	1.117*	1.907*	4.004*	3.034*	3.440*	4.292*	4.424*	3.697*
	<0.001	0.001	<0.001	<0.001	0.493	0.012	<0.001	<0.001	0.009	0.011	<0.001	<0.001	<0.001	<0.001	<0.001	0.001	0.001	0.001	0.001	0.004	0.035	<0.001	0.01	<0.001	<0.001	<0.001	<0.001
Observations	73	73	73	73	73	73	73	73	73	73	73	73	73	73	73	73	73	73	73	73	73	73	73	73	73	73	73
Adjusted R^2	-0.018	-0.037	0.013	-0.078	0.055	-0.02	-0.049	0.069	-0.109	-0.012	-0.054	-0.066	-0.019	0.071	0.055	0.007	0.071	0.024	-0.014	0.073	0.009	-0.046	0.027	-0.031	-0.062	-0.122	-0.077
F Statistic (df = 9; 63)	0.859	0.713	1.102	0.422	1.462	0.842	0.63	1.592	0.212	0.907	0.591	0.503	0.853	1.608	1.469	1.053	1.611	1.2	0.892	1.628	1.072	0.649	1.222	0.76	0.53	0.132	0.428

The first row is coefficient and the second row is p-value for each parameter.

All cytokines/chemokines were log(x+1) transformed. CEA, CA125 and CA19-9 were log(log(x+1)) transformed.

No adjustment for multiplicity was made due to the exploratory nature of our analysis.

*p<0.05

Supplementary Table 4B. Associations between cytokines and patient characteristics in CCC

	Cytokines/chemokines (coefficient and p-values)																										
	MIP-1β	IL-6	IFN-γ	IL-1Ra	IL-5	GM-CSF	TNF-α	Rantes	IL-2	IL-1β	Eotaxin	bFGF	VEGF	PDGF-BB	IP-10	IL-13	IL-4	MCP-1	IL-8	MIP-1α	IL-10	G-CSF	IL-15	IL-7	IL-12(p70)	IL-17	IL-9
Age	0.001	-0.013	0	-0.009	-0.009	-0.041	-0.002	-0.001	0	-0.008	-0.006	-0.01	-0.002	-0.031	0.005	0.001	-0.005	-0.008	0.001	0	0	0.002	-0.019	-0.011	0.007	-0.02	-0.001
	0.913	0.46	0.998	0.666	0.74	0.369	0.881	0.963	0.992	0.623	0.771	0.428	0.957	0.159	0.822	0.942	0.609	0.634	0.918	0.988	0.991	0.899	0.368	0.39	0.77	0.319	0.957
Menopause = Yes	0.098	0.288	-0.077	0.355	0.616	1.207	0.221	0.124	-0.193	0.409	0.082	0.366	0.106	0.32	-0.136	0.064	0.168	0.19	0.028	0.034	0.215	0.015	0.366	0.351	0.025	0.665	0.237
	0.648	0.42	0.853	0.379	0.247	0.176	0.5	0.748	0.675	0.211	0.851	0.146	0.85	0.451	0.757	0.807	0.36	0.547	0.897	0.813	0.489	0.95	0.383	0.176	0.958	0.097	0.592
Stage = III, IV	0.232	-0.384	-0.023	0.464	1.181	0.153	0.344	-0.255	-0.142	0.677	-0.094	0.277	-0.078	0.34	-0.757	0.265	0.325	-0.203	-0.271	-0.168	0.372	0.052	-0.326	0.364	0.314	0.767	0.427
	0.383	0.387	0.965	0.354	0.076	0.89	0.399	0.596	0.805	0.098	0.862	0.372	0.91	0.52	0.169	0.418	0.158	0.603	0.317	0.346	0.337	0.858	0.531	0.258	0.594	0.122	0.437
Ascites cytology = Positive	-0.128	-0.007	0.149	0.082	-0.105	-0.04	0.006	0.068	0.107	-0.04	0.095	-0.039	-0.319	-0.086	0.08	-0.191	0.007	0.105	-0.01	0.011	-0.35	0.074	0.39	-0.088	-0.434	-0.084	-0.35
	0.358	0.976	0.576	0.754	0.76	0.945	0.98	0.786	0.722	0.85	0.736	0.812	0.377	0.756	0.78	0.263	0.95	0.608	0.945	0.907	0.087	0.626	0.154	0.6	0.162	0.744	0.225
Peritoneal dissemination = Yes	-0.227	0.843*	0.287	-0.141	-0.914	0.883	-0.023	-0.418	0.486	-0.358	0.284	-0.172	0.137	-0.577	1.122*	0.082	-0.312	0.188	0.537*	0.013	-0.13	0.072	0.603	-0.106	-0.213	-0.8	-0.058
	0.367	0.048	0.553	0.765	0.144	0.397	0.952	0.358	0.371	0.35	0.577	0.557	0.833	0.249	0.033	0.791	0.151	0.61	0.039	0.94	0.721	0.792	0.222	0.725	0.703	0.089	0.911
Lymph node metastasis = Yes	0.219	0.73	0.256	0.222	-0.319	1.705	0.108	0.055	0.739	0.089	-0.145	0.484	0.406	0.456	-0.393	0.03	0.128	0.244	0.525	0.409*	-0.152	0.391	0.808	0.103	0.294	0.797	0.64
	0.453	0.137	0.648	0.685	0.658	0.16	0.809	0.917	0.242	0.841	0.807	0.157	0.591	0.43	0.512	0.932	0.608	0.569	0.079	0.04	0.719	0.22	0.159	0.769	0.649	0.143	0.289
CEA	-0.081	0.012	-0.095	0.079	-0.047	0.013	-0.147	-0.185	-0.126	-0.055	0.184	-0.249	-0.04	-0.129	0.14	-0.123	-0.239*	0.211	-0.146	-0.225*	-0.075	-0.196	0.389	-0.096	-0.305	-0.453	-0.223
	0.518	0.954	0.695	0.739	0.88	0.981	0.446	0.418	0.642	0.772	0.474	0.094	0.904	0.606	0.588	0.425	0.031	0.256	0.256	0.01	0.683	0.156	0.118	0.529	0.278	0.056	0.393
CA125	0.516*	0.991*	0.324	0.458	0.754	-0.081	0.521	0.315	0.095	0.63	-0.007	0.35	1.164*	0.188	0.29	0.708*	0.208	0.127	0.397	0.077	1.030**	0.127	0.153	0.617*	1.205*	0.267	0.416
	0.022	0.009	0.445	0.27	0.169	0.929	0.126	0.429	0.842	0.064	0.989	0.176	0.046	0.668	0.523	0.011	0.272	0.696	0.079	0.604	0.002	0.598	0.722	0.023	0.017	0.512	0.362
CA19-9	-0.063	0.226	-0.167	-0.148	0.104	0.063	-0.144	0.014	-0.104	-0.077	-0.115	0.093	0.033	0.044	-0.163	-0.107	-0.007	-0.009	0.134	0.038	-0.009	0.024	-0.123	-0.069	-0.179	0.119	0.101
	0.584	0.24	0.452	0.494	0.713	0.895	0.413	0.945	0.674	0.658	0.622	0.486	0.914	0.847	0.49	0.448	0.943	0.959	0.253	0.619	0.956	0.848	0.583	0.619	0.483	0.575	0.671
Constant	3.816*	1.197	5.098*	4.488*	1.264	2.246	3.907*	9.429*	1.466	0.765	5.694*	3.383*	2.935*	9.542*	7.008*	1.545*	2.132*	3.403*	2.100*	1.396*	1.249	3.711*	1.139	2.672*	2.500*	4.330*	3.181*
	<0.001	0.173	<0.001	<0.001	0.33	0.301	<0.001	<0.001	0.197	0.337	<0.001	<0.001	0.034	<0.001	<0.001	0.019	<0.001	<0.001	0.001	0.001	0.104	<0.001	0.268	<0.001	0.035	<0.001	0.005
Observations	66	66	66	66	66	66	66	66	66	66	66	66	66	66	66	66	66	66	66	66	66	66	66	66	66	66	66
Adjusted R^2	0.052	0.304	-0.041	0	0.05	0.026	0.045	-0.054	-0.027	0.118	-0.126	0.141	-0.023	-0.015	0.008	0.124	0.071	-0.105	0.256	0.086	0.165	0.032	0.052	0.126	0.026	0.134	0.017
F Statistic (df = 9; 56)	1.399	4.153*	0.713	1.002	1.382	1.193	1.338	0.633	0.811	1.969	0.195	2.187*	0.839	0.89	1.061	2.022	1.548	0.314	3.479*	1.676	2.429*	1.241	1.394	2.038	1.191	2.113*	1.124

The first row is coefficient and the second row is p-value for each parameter.

All cytokines/chemokines were log(x+1) transformed. CEA, CA125 and CA19-9 were log(log(x+1)) transformed.

No adjustment for multiplicity was made due to the exploratory nature of our analysis.

*p<0.05

Supplementary Table 4C. Associations between cytokines and patient characteristics in EMC

	Cytokines/chemokines (coefficent and p-values)																										
	MIP-1β	IL-6	IFN-γ	IL-1Ra	IL-5	GM-CSF	TNF-α	Rantes	IL-2	IL-1β	Eotaxin	bFGF	VEGF	PDGF-BB	IP-10	IL-13	IL-4	MCP-1	IL-8	MIP-1α	IL-10	G-CSF	IL-15	IL-7	IL-12(p70)	IL-17	IL-9
Age	0.016	0.009	-0.007	-0.002	-0.016	-0.014	-0.009	0.014	0.003	-0.007	0.013	0.01	0.037	0.005	0.011	-0.001	-0.009	0.004	-0.011	-0.008	0.012	-0.003	0.019	0.004	0.014	0.004	-0.007
	0.172	0.529	0.613	0.926	0.246	0.654	0.42	0.375	0.892	0.398	0.187	0.539	0.188	0.806	0.499	0.946	0.405	0.779	0.377	0.288	0.658	0.787	0.42	0.773	0.551	0.843	0.603
Menopause = Yes	-0.751*	-0.455	-0.245	-0.296	0.102	0.112	-0.091	-0.355	-0.612	-0.009	-0.258	-0.338	-1.043	-0.477	0.182	-0.269	-0.039	-0.442	0.065	0.061	-0.873	-0.204	-0.793	-0.365	-0.63	-0.386	0.002
	0.037	0.289	0.554	0.642	0.802	0.907	0.786	0.444	0.404	0.971	0.4	0.473	0.221	0.421	0.705	0.564	0.901	0.333	0.857	0.776	0.296	0.548	0.263	0.395	0.381	0.533	0.997
Stage = III, IV	0.204	-0.085	-0.829	-0.613	-0.895*	-1.339	-0.585	-0.47	-0.5	-0.468	-0.171	-1.504*	-0.693	-0.894	0.378	-0.858	-0.508	-0.101	0.154	-0.554*	-0.796	-0.780*	0.533	-0.691	-0.53	-2.022*	-0.815*
	0.563	0.844	0.056	0.347	0.038	0.177	0.092	0.322	0.501	0.07	0.582	0.004	0.421	0.144	0.441	0.077	0.12	0.828	0.674	0.017	0.348	0.03	0.457	0.12	0.467	0.003	0.05
Ascites cytology = Positive	0.342	0.347	0.361	0.3	0.253	1.22	0.342	0.137	0.404	0.243	0.125	0.617*	0.289	0.361	-0.166	0.393	0.185	-0.02	0.09	0.274	0.361	0.398	0.159	0.229	0.414	0.856*	0.513*
	0.131	0.21	0.18	0.464	0.337	0.055	0.117	0.644	0.39	0.132	0.527	0.048	0.594	0.345	0.59	0.194	0.361	0.946	0.696	0.054	0.497	0.075	0.724	0.406	0.369	0.038	0.05
Peritoneal dissemination = Yes	0.109	0.271	0.439	0.527	0.375	1.424*	0.408	0.205	0.498	0.298	0.085	0.524	0.372	0.019	0.275	0.385	0.236	0.092	0.202	0.319*	0.53	0.391	0.272	0.366	0.461	0.867*	0.384
	0.649	0.36	0.132	0.237	0.19	0.039	0.085	0.523	0.328	0.089	0.688	0.115	0.525	0.963	0.41	0.237	0.283	0.769	0.42	0.039	0.358	0.104	0.577	0.223	0.354	0.05	0.168
Lymph node metastasis = Yes	-0.408	-0.483	0.442	0.107	0.67	-1.014	0.323	0.185	-0.169	0.223	0.084	0.936	0.031	0.549	-0.38	0.422	0.267	-0.227	-0.245	0.418	-0.017	0.508	-1.405	0.319	-0.041	1.128	0.361
	0.295	0.313	0.341	0.88	0.148	0.347	0.387	0.721	0.836	0.419	0.806	0.082	0.974	0.407	0.479	0.417	0.45	0.654	0.543	0.089	0.985	0.186	0.081	0.505	0.959	0.11	0.416
CEA	0.215	-0.258	-0.04	-0.218	0.099	-0.647	-0.048	-0.225	-0.142	-0.014	-0.134	-0.381	-0.404	-0.328	-0.052	0.023	0.071	0.163	0.15	-0.036	-0.24	-0.031	-0.093	-0.069	-0.23	-0.361	0.023
	0.208	0.22	0.843	0.482	0.617	0.173	0.768	0.323	0.69	0.906	0.37	0.104	0.328	0.26	0.826	0.919	0.645	0.463	0.394	0.73	0.552	0.85	0.786	0.742	0.509	0.235	0.906
CA125	-0.503	0.883*	0.129	0.622	0.247	1.23	0.141	-0.169	0.744	0.153	0.271	0.705	0.978	0.152	0.596	0.354	0.168	0.174	0.401	0.162	0.609	0.395	0.591	0.218	0.586	0.566	0.206
	0.144	0.041	0.75	0.321	0.537	0.197	0.667	0.71	0.301	0.527	0.368	0.133	0.241	0.793	0.21	0.438	0.586	0.695	0.259	0.443	0.454	0.24	0.392	0.602	0.404	0.353	0.596
CA19-9	-0.069	-0.358	-0.429*	-0.580*	-0.436*	-1.341*	-0.317*	-0.11	-0.748*	-0.293*	-0.178	-0.506*	-0.438	-0.38	-0.128	-0.4	-0.262	-0.299	-0.089	-0.168	-0.665	-0.387*	-0.492	-0.346	-0.356	-0.561*	-0.196
	0.648	0.063	0.024	0.045	0.02	0.004	0.036	0.589	0.025	0.011	0.19	0.019	0.24	0.149	0.545	0.057	0.065	0.139	0.573	0.083	0.074	0.014	0.117	0.072	0.26	0.045	0.262
Constant	4.967*	1.121	6.408*	4.694*	2.649*	0.997	5.076*	9.565*	1.723	1.624*	4.804*	2.684*	2.407	8.961*	6.567*	2.618*	2.835*	3.627*	3.050*	1.869*	2.476	4.219*	-0.177	3.110*	3.563*	3.641*	3.712*
	<0.001	0.18	<0.001	0.001	0.002	0.592	<0.001	<0.001	0.227	0.002	<0.001	0.006	0.147	<0.001	<0.001	0.007	<0.001	0.001	0.001	<0.001	0.129	<0.001	0.897	0.001	0.015	0.005	<0.001
Observations	39	39	39	39	39	39	39	39	39	39	39	39	39	39	39	39	39	39	39	39	39	39	39	39	39	39	39
Adjusted R^2	0.032	0.245	0.052	-0.006	0.061	0.305	0.049	-0.148	0.041	0.085	-0.047	0.201	-0.027	0.007	0.111	-0.022	-0.041	-0.133	0.058	0.104	-0.033	0.138	0.089	-0.049	-0.091	0.148	-0.01
F Statistic (df = 9; 29)	1.141	2.373*	1.233	0.975	1.276	2.850*	1.216	0.457	1.18	1.39	0.81	2.063	0.888	1.031	1.529	0.908	0.833	0.504	1.261	1.489	0.866	1.676	1.414	0.802	0.649	1.733	0.957

The first row is coefficient and the second row is p-value for each parameter.

All cytokines/chemokines were log(x+1) transformed. CEA, CA125 and CA19-9 were log(log(x+1)) transformed.

No adjustment for multiplicity was made due to the exploratory nature of our analysis.

*p<0.05

Supplementary Table 5. Univariate and Multivariate analyses for prognostic outcomes of CCC

Histotype	Outcome	Cluster	Univariate analysis		Multivariate analysis*	
			Hazard ratio (95% CI)	*P* -value	Hazard ratio (95% CI)	*P* -value
Clear cell	Progression-free survival	$C1^{lo}C2^{lo}$	Ref	0.0018	Ref	0.028
		$C1^{lo}C2^{hi}$	3.12 (0.56, 17.20)		1.52 (0.25, 9.20)	
		$C1^{hi}C2^{lo}$	4.90 (1.04, 23.12)		3.70 (0.76, 18.06)	
		$C1^{hi}C2^{hi}$	11.49 (2.51, 52.56)		5.66 (1.15, 27.76)	
	Overall survival	$C1^{lo}C2^{lo}$	Ref	0.035	Ref	0.21
		$C1^{lo}C2^{hi}$	1.42 (0.20, 10.19)		0.55 (0.07, 4.56)	
		$C1^{hi}C2^{lo}$	2.74 (0.53, 14.11)		1.40 (0.24, 8.05)	
		$C1^{hi}C2^{hi}$	7.04 (1.46, 33.99)		2.61 (0.47, 14.49)	

* Adjusted by age and stage

Supplementary Table 6. The correlations between serum cytokine level and local cytokine expression in CCC

	Group number of the serum cytokine signature	Cytokine Protein level Average	Cytokine mRNA expression Average	Pearson's correlation coefficient Rho	Pearson's correlation coefficient P-value
MIP1β	Signature 1	4.632671137	5.806984	0.397820009	0.00422329
IL6	Signature 1	2.638200422	7.565104	0.277114126	0.051382158
IFNγ	Signature 1	5.542068159	0.6664288	-0.096389352	0.505481559
IL1Ra	Signature 2	4.873180573	3.546568	-0.201088025	0.161425474
IL5	Signature 2	2.467042613	0	NA*	NA*
GMCSF	Signature 1	0.978781092	0.034732	-0.076531031	0.597329643
TNFα	Signature 2	4.664454722	4.436118	0.021336526	0.883074639
Rantes	Signature 1	9.894347945	6.787804	0.025607116	0.859886423
IL2	Signature 1	1.533390671	0	NA*	NA*
IL1β	Signature 2	1.522645244	4.373468	0.04629525	0.749538238
Eotaxin	Signature 1	5.312174219	1.0312848	0.139432551	0.33418025
bFGF	Signature 2	3.799539775	0	NA*	NA*
VEGF	Signature 1	4.659050497	10.087036	0.012144924	0.93328818
PDGFBB	Signature 1	8.384423717	4.791672	0.082205481	0.570347174
IP10	Signature 1	7.575041176	1.934582	0.019176036	0.894843864
IL13	Signature 2	2.618465329	0	NA*	NA*
IL4	Signature 2	2.372353809	0	NA*	NA*
MCP1	Signature 1	3.325954658	7.733904	-0.047503437	0.743222315
IL8	Signature 1	3.082205964	8.175506	0.095007255	0.511632425
MIP1α	Signature 2	1.631987874	6.6803	0.000521851	0.997130246
IL10	Signature 2	2.8929473	1.7355238	0.091816545	0.525975379
GCSF	Signature 2	4.172151426	0.069052	0.225191033	0.115872346
IL15	Signature 1	0.58640387	4.797966	-0.209580191	0.144083742
IL7	Signature 2	3.205420823	0.24124	0.050926049	0.72542212
IL12(p70)	Signature 1	4.430029111	0	NA*	NA*
IL17	Signature 2	4.32105836	0.05023	0.184192292	0.200375576
IL9	Signature 2	4.093817151	0	NA*	NA*

NA* : Not computable due to no expression

FGF is not included in any of the panels and PDGF is from IO360.
